# Blind Quality Assessment of Images Containing Objects of Interest

**DOI:** 10.3390/s23198205

**Published:** 2023-09-30

**Authors:** Wentong He, Ze Luo

**Affiliations:** 1 Computer Network Information Center, Chinese Academy of Sciences, Beijing 100190, China; hwt0316@cnic.cn; 2 University of Chinese Academy of Sciences, Beijing 100049, China

**Keywords:** blind image quality assessment, objects of interest, object detection, transformer, DETR, multi-task

## Abstract

To monitor objects of interest, such as wildlife and people, image-capturing devices are used to collect a large number of images with and without objects of interest. As we are recording valuable information about the behavior and activity of objects, the quality of images containing objects of interest should be better than that of images without objects of interest, even if the former exhibits more severe distortion than the latter. However, according to current methods, quality assessments produce the opposite results. In this study, we propose an end-to-end model, named DETR-IQA (detection transformer image quality assessment), which extends the capability to perform object detection and blind image quality assessment (IQA) simultaneously by adding IQA heads comprising simple multi-layer perceptrons at the top of the DETRs (detection transformers) decoder. Using IQA heads, DETR-IQA carried out blind IQAs based on the weighted fusion of the distortion degree of the region of objects of interest and the other regions of the image; the predicted quality score of images containing objects of interest was generally greater than that of images without objects of interest. Currently, the subjective quality score of all public datasets is in accordance with the distortion of images and does not consider objects of interest. We manually extracted the images in which the five predefined classes of objects were the main contents of the largest authentic distortion dataset, KonIQ-10k, which was used as the experimental dataset. The experimental results show that with slight degradation in object detection performance and simple IQA heads, the values of PLCC and SRCC were 0.785 and 0.727, respectively, and exceeded those of some deep learning-based IQA models that are specially designed for only performing IQA. With the negligible increase in the computation and complexity of object detection and without a decrease in inference speeds, DETR-IQA can perform object detection and IQA via multi-tasking and substantially reduce the workload.

## 1. Introduction

In the real world, in order to monitor objects of interest, a large number of image-capturing devices, such as camera traps [1,2,3] for observing wild animals and video surveillance cameras for recording the activities of people, have been deployed. These automatic recording cameras produce a large number of images containing objects of interest at all times. These images can provide sufficient and diverse image data for many applications and research studies [4,5]. For example, the images from camera traps have recorded various animal behaviors without human interference, and people can use these images for animal protection and conduct various animal behavior analyses [6,7,8,9]. Thus, image quality assessment (IQA) plays an important role in image applications because the images can undergo various distortions during image acquisition, compression, transmission, etc. [9]. Since people are the final users of images, people’s subjective assessment of an image’s quality is the most direct and reliable method [10]. However, the number of images substantially exceeds subjective human assessment capacities. Thus, objective image quality assessments, which enable computers to make assessments that are consistent with subjective assessments, have recently attracted increasing attention [11,12,13].

Generally, IQA methods can be categorized as full-reference IQA [14,15], reduced-reference IQA [16,17], and no-reference or blind IQA [18,19] via the availability of reference images. Full-reference IQA and reduced-reference IQA methods, respectively, utilize full or a part of the information from reference images to assess the distorted image’s quality. Since they do not require reference images, which are usually difficult to obtain or completely unavailable in real-world applications, blind IQA methods are more challenging but more applicable [20,21].

In recent years, due to the achieved remarkable results of deep learning [22] in many computer vision tasks, many deep learning-based blind IQA models have been proposed [23,24,25,26,27,28,29]. Deep learning-based blind IQA methods have significantly outperformed other types of methods, such as natural scene statistic-based methods [12,30,31] and texture feature-based methods [32,33], which have currently become the dominant scheme [21]. Based on the assumption that the high-level semantic features extracted by pre-trained CNN (convolutional neural network) models for image classification tasks on large-scale datasets (such as ImageNet [34] and Places365 [35]) are quality-aware, many proposed CNN-based methods leverage these features to predict the subjective quality score, and this is carried out using mainly two methods: using a regression model such as support vector regression to regress the features onto the subjective score, or replacing the final classification layer with a regression layer and fine-tuning the regression layer with the IQA datasets to predict the image quality score [23,36,37,38]. The latter method results in end-to-end image quality assessments. Li et al. [9] utilized three well-known pretrained classification deep CNNs, including AlexNet [39] and ResNet [40], to extract deep semantic features and trained a linear regression model for regressing the aggregated feature onto the image quality score. Li et al. [41] utilized a network in network (NiN) model that was first pretrained on ImageNet to extract features and fine-tuned the concatenated new layers to map the learned features onto the subjective quality score. Finally, the model could directly take raw images as inputs and estimate the image quality. Very recently, end-to-end deep learning-based blind IQA methods have become the mainstream methods. Ma et al. [42] proposed a multi-task end-to-end CNN-based blind IQA method, namely MEON, which consists of two sub-networks for identifying the distortion type and predicting the quality score. Rehman et al. [43] proposed an end-to-end deep learning-based region and proposed the network (RPN) methodology for blind IQA, which used pretrained VGGNet and ResNet to extract feature maps, and RPN was used to extract region proposals from feature maps as the regions of interest (ROIs); moreover, fully connected and regression layers were used to compute the local quality score of ROI, and the average of all local ROI scores was used as the final total image quality score. For the first time, they leveraged the proposals to predict image quality and achieved standout accuracies with respect to synthetically distorted and real-world images.

It is well known that the receptive field of CNN is limited and only captures the local features of the image, thus losing non-local information and having a strong local bias. Moreover, due to the spatial invariance of shared convolution kernel weights, CNN has limited abilities in handling complex feature combinations. The attention mechanisms of the transformer [44] can aggregate global information from entire images [38]. In various computer vision tasks, such as classification [45] and object detection [46], transformer has achieved great success. In the real world, authentic images, especially the images captured from the wild, not only contain global distortions caused by low illumination, loss of focus, fog, rain, etc., but also suffer from distortions in local areas, which are caused by an object’s fast movement, overexposure, etc. Thus, deep blind IQA models should accurately capture local and global features in order to fuse them and carry out image quality prediction [27]. Following the utilization of the transformer encoder in vision transformer model [45], You et al. [47] proposed an architecture that leverages the feature maps extracted by CNN as input to the transformer encoder for image quality assessments. To address the shortcoming of image quality degradation caused by resizing and cropping images to fixed shapes in CNN-based models, Ke et al. [48] designed a multi-scale image quality transformer (MUSIQ) that encodes multi-aspect ratio multi-scale image features into a sequence of tokens as the input of the transformer encoder for image quality score prediction. Golestaneh et al. [38] sequenced the extracted features into the transformer encoder and applied relative ranking and self-consistency loss to reduce the uncertainty of the model. Yang et al. [49] first applied attention mechanisms across the channel and spatial dimensions on the extracted features via the vision transformer model to predict the quality score, and they achieved state-of-the-art performance.

**Figure 1:**
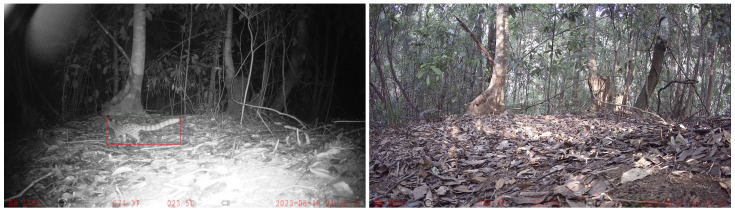
A real-world example of an image containing animals and a blank image without animals. The left image should be more valuable and is of better quality than the right image because it captures Prionailurus bengalensis (marked in red rectangle) while exhibiting more severe distortion, such as dimming and overexposure, than the right image.

In fact, the devices for monitoring objects of interest inevitably take on a large number of blank background images that do not contain objects of interest, such as animals. Currently, most deep learning-based blind IQA methods only evaluate the image’s quality according to the distortion, and they do not consider the content of the objects of interest. When the distortion degree of the images containing objects of interest is greater than that of the images without objects of interest, the quality assessment predicted via the blind IQA of the former is worse than the latter, which is unreasonable or even incorrect in some real-world applications. For example, as shown in Figure 1, although the left image, which records the nocturnal behavior of animals, exhibits more severe distortion, such as dimming and overexposure, than the right image without animals, which was taken during the day, the left image is more valuable and exhibits better quality than the right image due to the presence of animals. Thus, we present a practical problem with respect to the blind IQA: how do we objectively and accurately evaluate the quality of images containing objects of interest?

For obtaining accurate image quality assessments, humans should first focus on the image’s content, especially the objects of interest. Moreover, the human visual system first pays attention to the objects of interest in an image and perceives its quality. Cao et al. [50] used an object detector to detect objects and aggregated the features of the detected objects and the image to assess image quality. The achieved state-of-the-art performance demonstrated that the objects in the images are highly relevant for IQA. Inspired by this work, the object detection network should have the potential to solve the above practical problem. In practical applications, the most direct way to blindly assess the quality of images containing objects of interest is to first filter out these images using a deep object detection model and then carry out a quality assessment on these images. Can a deep learning-based model perform both object detection and blind image quality assessment simultaneously in a multi-tasking manner (thus simplifying the process and reducing efforts, which renders it more suitable for practical application requirements)? Thus far, no research conducted has met the above requirements.

In this paper, we chose to use an advanced object detection model, DETR (detection transformer) [46], as the baseline model. Referring to the object detection heads of DETR, we added blind IQA heads on the top of the transformer decoder to extend its capability to perform object detection and blind IQA simultaneously; moreover, we named the final model DETR-IQA. The blind IQA heads consisting of simple multilayer perceptron (MLP) [51] translate the object queries from the decoder of DETR into image quality scores, and we set a hyperparameter to linearly combine the L2 loss of objects and no objects with a subjective score. We transformed the traditional blind IQA based on the distortion degree of the entire image into the blind IQA based on the weighted fusion of the distortion degree of the region of objects of interest and the other regions of the image. Finally, the proposed DETR-IQA can not only carry out accurate blind quality assessments of images containing objects of interestbut it can also carry out realistic and reasonable quality assessments relative to images without objects of interest. In addition, DETR-IQA performs object detection and blind IQA simultaneously with negligible increases in model computation and complexity, which can substantially reduce the practical workload.

The structure of this paper is as follows: In Section 2, we present the materials and methods used in this study. The details of images containing objects of interest and the results of several experiments are presented in Section 3. In Section 4, we discuss the results presented in Section 3 and the deficiencies and direction of our work. In Section 5, we present the conclusions.

## 2. Materials and Methods

### 2.1. Images Containing Objects of Interest

**Figure 2:**
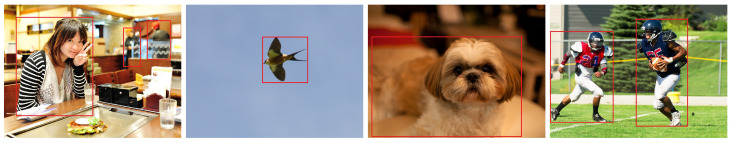
Example of images containing objects of interest, which are marked in rectangles and are the main content components of the image.

Several commonly used public datasets for image quality assessment can roughly be categorized as synthetic and authentic [21] datsets. Synthetic datasets, such as LIVE and TID2013 datasets, consist of reference images and distorted reference images of several types, such as JPEG2000 compression, white noise, Gaussian blur, and fast fading [52,53] distortion types in synthetic images could occur in real-world image applications, artificially synthesizing some complex distortions, such as blur, which is caused by the fast motion of objects, is difficult [54]. In this study, we worked on the blind IQA of real-world images containing objects of interest. Thus, we selected some images from the largest authentic dataset, KonIQ-10k [54], which contains 10,073 quality score images, with each image scoring a reliable 120 in terms of ratings from 1459 crowd workers on a crowdsourcing platform; these were used to construct the experimental dataset. The subjective score is in the form of a mean opinion score (MOS) ranging from 1 to 100, in which the larger MOS values present better image quality. The subjective quality score of images in KonIQ-10k is mainly used to consider 8 distortion types: noise, JPEG artifacts, aliasing, lens and motion blur, over-sharpening, wrong exposure, color fringing, and over-saturation. The crowd workers carried out image quality assessment based on the degree of the above types of distortion, and they were not instructed to consider the content of some classes of objects, such as people, birds, dogs, etc., in the images. Thus, before selecting the images, we firstly predefined some classes of objects of interest and then manually extracted the images relative to which the objects of interest were the main content component. The object of interest as the main content component can ensure that the MOS score of the image is mainly based on the object of interest. Some examples of images containing objects of interest are shown in Figure 2. The predefined class of objects of interest comprised people, birds, dogs, horses, and other animals, including sheep, animal sheer, monkey, etc. The total number of images is 3582, in which the number of images of people is 2069, bird images number 543, dog images number 456, horse images number 105, and other animal images number 541. Finally, we used a labeling tool to annotate images with a bounding box with respect to the objects of interest. We also randomly extracted 460 images without objects of interest to verify whether the proposed DETR-IQA can carry out reasonable and realistic image quality prediction: that is, it does not exhibit the problems discussed in the Introduction.

### 2.2. DETR-IQA

In real-world image applications, even if the distortion of the images containing objects of interest, such as rare animals, is more severe than that of the images without objects of interest, they are more valuable and exhibit better quality because they record the behavior and activity of the objects of interest. Therefore, people are more concerned about the distortion degree of the objects of interest in images. The human visual system first pays attention to the objects of interest in an image and perceives its quality. Since the behavior of the object of interest in the image is closely related to its surrounding environment, the image’s background information can be used as auxiliary information for IQA. To mimic the above process of a human being perceiving the image’s quality, the blind IQA model can be divided into two stages: object detection for extracting the features of the objects of interest and image quality assessment for predicting the quality score based on the extracted features.

**Figure 3:**
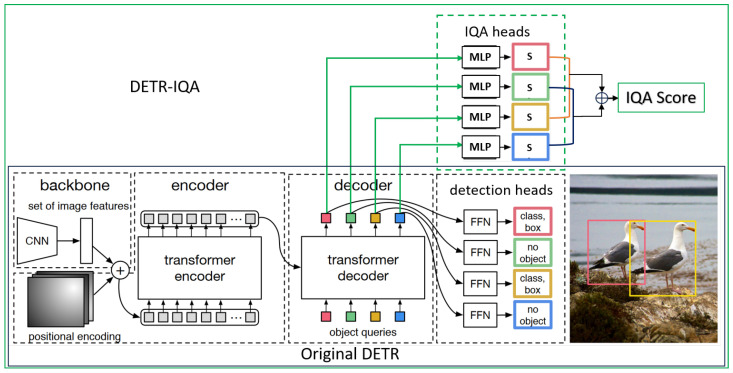
An overview of DETR-IQA, in which the bottom is the original DETR model and DETR-IQA is the DETR with IQA heads.

Transformer, which solely relies on the attention mechanism, was first proposed in NLP tasks [44], and it achieved great performance with respect to various compute vision tasks. In the object detection task, DETR (detection transformer) [46] employs the pure transformer architecture to model object detection as a set prediction task without using hand-designed components, such as anchor design and non-maximum suppression. The multi-scale features of input images are first extracted by CNN and then fed into the encoder together with positional embedding. DETR uses a fixed number of learnable queries to probe the outputs of an encoder in a decoder and then adds a feedforward network on top of the decoder to predict either an object (object class with bounding box) or no-object (background class). DETR simplifies the detection pipeline without the need for hand-designed anchors [55] and non-maximum suppression [56], and it predicts all objects at once. The architecture of DETR is simple yet effective in the object detection task. The fixed number of decoder outputs contains the features of the objects of interest and can be used to assess the image’s quality. Another advantage of DETR is that it is easy to expand because of its simple and advanced architecture. Thus, we extended the capabilities of DETR by adding IQA heads to carry out image quality assessment without degrading the object detection performance, and we named this model DETR-IQA. To the best of our knowledge, this is the first attempt to blindly assess image quality based on objects of interest. As a DETR-like model, DETR-IQA consists of four main components: a multi-scale CNN backbone, multi-layer transformer encoder–decoder, simple feedforward network (FFN) with a linear projection as detection heads, and simple multilayer perceptron (MLP) as IQA heads. In this study, the feedforward network and multilayer perceptron have the same structure, and only the dimensions of the outputs differ. The overall network is illustrated in Figure 3.

Given an input image, a conventional CNN backbone, such as ResNet-50, extracts multi-scale features. Flattened features supplemented with fixed positional encodings as a sequence are fed into the transformer encoder. The features from the encoder and N (e.g., N = 100) object queries, which are learnable positional embeddings, are fed into the transformer decoder, which contains cross-attention and self-attention modules. Then, the feedforward network regresses the object queries produced by the decoder relative to the bounding box coordinates, and a linear projection generates the classification results. Because the number of object queries is greater than the actual number of objects, the object queries not match the ground truth are denoted by the “no-object” class label. We intuitively think that the features of no objects contain some information in the background: more precisely, the surrounding information of objects that can assist the IQA. The multi-layer perceptron of IQA heads translates all object queries from the decoder into quality scores; then, all scores are averaged in some way as the image quality score. DETR-IQA is completely inherited from the original DETR and is then leveraged on the object queries of the DETR decoder to convert the IQA; this is carried out based on the distortion of the entire image relative to the IQA’s object and no-object distortion. In addition, via multi-tasking, DETR-IQA can perform object detection and blind IQA simultaneously, which simplifies the practical process and reduces the practical workload.

The loss of DETR-IQ contains two parts—original loss from DETR and designed loss relative to the blind IQA head—and is defined as follows:

(1) $$L_{DETR-IQA}=L_{DETR}+L{}_{IQA}$$

where $L_{DETR}$ defines a linear combination of standard cross-entropy functions for classification and a combination of absolute error (L1 loss) and generalized IoU (intersection over union) for box coordinate prediction, and $L_{IQA}$ defines a linear combination of object score loss and no-object score loss. For a more detailed description and details of the loss from DETR, we refer the reader to the DETR literature. Partly because of the blind IQA loss, we first caculated the L2 loss for all matched object regression scores with an averaged and partial MOS and all unmatched no-object regression scores with an averaged and partial MOS; finally, we added the two L2 losses as the image quality assessment’s loss. Herein, the function is as follows:

(2) $$L{}_{IQA}=L2\left(\frac{\sum_{i=1}^{O}S_{i}}{O},\lambda *MOS\right)+L2\left(\frac{\sum_{j=1}^{NO}S_{j}}{NO},\left(1-\lambda \right)*MOS\right)$$

where $S_{i}$ and $S_{j}$, respectively, represent the score of one object and one no object, O represents the number of matched objects and NO represents the number of unmatched no objects. The sum of O and NO is equal to the fixed number of learnable quries. We intuitively considered that the MOS of an image consists of the MOS of objects and no objects, and we used λ as the hyperparameter to linearly split the MOS into two parts. We determined the value of λ based on the experimental results of object detection and image quality assessments.

### 2.3. Evaluation Metrics

Following prior studies, we selected Spearman’s rank-order correlation coefficient (SRCC) and Pearson’s linear correlation coefficient (PLCC) to evaluate the performance of DETR-IQA. The metric used to evaluate the performance of DETR-IQA relative to object detection comprises the traditional and commonly used average precision at different IoUs.

The SRCC calculates the monotonic relationship between the subjective and predicted scores and is defined as follows:

(3) $$SRCC=1-\frac{6\sum_{i}^{}d_{i}^{2}}{N(N^{2}-1)}$$

where $d_{i}$ represents the rank difference between the MOS and the predicted score of the *i*-th image, and *N* represents the number of test images.

The PLCC computes the linear correlation between the MOS values and predicted scores, and it is defined as follows:

(4) $$PLCC=\frac{\sum_{i}\left(s_{i}-m_{s_{i}}\right)\left({\hat{s}}_{i}-m_{{\hat{s}}_{i}}\right)}{\sqrt{\sum_{i}{\left(s_{i}-m_{s_{i}}\right)}^{2}}\sqrt{\sum_{i}{\left({\hat{s}}_{i}-m_{{\hat{s}}_{i}}\right)}^{2}}}$$

where $s_{i}$ and ${\hat{s}}_{i}$, respectively, represent the MOS and predicted scores of the *i*-th test image, and $m_{i}$ and ${\hat{m}}_{i}$, respectively, represent the mean of the MOS and the predicted scores of all test images.

When values of SRCC and PLCC are closer to 1, this indicates the better performance of the blind IQA.

### 2.4. Implementation Details

In this study, we implemented the DETR-IQA model via PyTorch, and experiments were run on 2 NVIDIA TITAN RTX GPUs, each with 24G VRAM size. We trained 300 epochs with a batch size of 2. Moreover, we leveraged the pre-trained DETR model with the ResNet-50 backbone on COCO 2017 val5k and fine-tuned it using our constructed dataset. We did not change the structure of the baseline DETR model and used the default parameters. DETR-IQA is composed of ResNet-50, a 6-layer transformer encoder, and a 6-layer transformer decoder. We used an AdamW optimizer with a weight decay of 1 $\times \;10^{-4}$ and at most 200 epochs. We set the initial transformer’s learning rate to 1 $\times \;10^{-4}$ and the backbone’s learning rate to 1$\times \;10^{-5}$. The weights of DETR-IQA were initialized with a COCO-pretrained DETR model. The hyperparameters λ were empirically set to 0.99, 0.9, 0.8, 0.7, and 0.6. According to the ratio of 8:2, the constructed images containing objects of interest were randomly divided into the training set and testing set.

## 3. Results

### 3.1. Images Containing Objects of Interest

**Figure 4:**
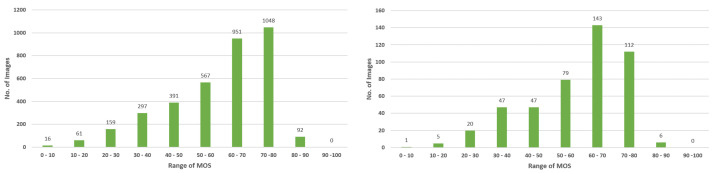
The distribution histogram of the MOS of images containing objects of interest (**left**) and images without objects of interest (**right**).

In some real-world applications, especially monitoring objects of interest such as animals, it is common that the images containing objects of interest, such as nocturnal animals and rare animals, exhibit greater distortion than images without objects of interest. It is unreasonable or even incorrect to assess the images’ quality only based on image distortion because people subjectively think that images containing objects of interest are more valuable and of better quality than images without objects of interest and, therefore, should have greater quality scores. In order to simulate the images’ data in real-world applications, we predefined five classes of objects of interest and extracted images in which the objects of interest are the main content components in KonIQ-10k. The object of interest occupying the major region of an image can ensure that the subjective quality score is mainly based on the perception of objects of interest. Then, we manually annotated the bounding box of the objects in each image. At the same time, we also randomly selected 460 images without objects of interest to evaluate the performance of the proposed model relative to images without objects of interest. The details of images containing objects of interest are shown in Table 1. The distribution histogram of the MOS of these extracted images is shown in Figure 4.

According to the ratio of 8:2, the images containing objects of interest were randomly divided into the training set and testing set, as shown in Table 2.

### 3.2. Performance Evaluation

In this study, we added the MLP on top of the transformer decoder to extend the capability of DETR to perform object detection and blind IQA via multi-tasking. The IQA heads and detection heads shared the same backbone: the transformer encoder and transformer decoder. Because the ground-truth image quality score’s MOS did not contain any information about the location of objects in the image, the IQA heads could degrade the performance of object detection. However, the IQA heads depend on the outputs of the decoder; thus, the object detection performance of DETR-IQA should not degrade too much and should preferably be close to the performance of the baseline DETR model.

We firstly conducted a DETR experiment without using IQA heads to provide the baseline object detection performance as shown in Table 3.

Then, we conducted several DETR-IQA experiments using different hyperparameters λ to find the suitable value. The results of the experiments are shown in Table 4.

**Figure 5:**
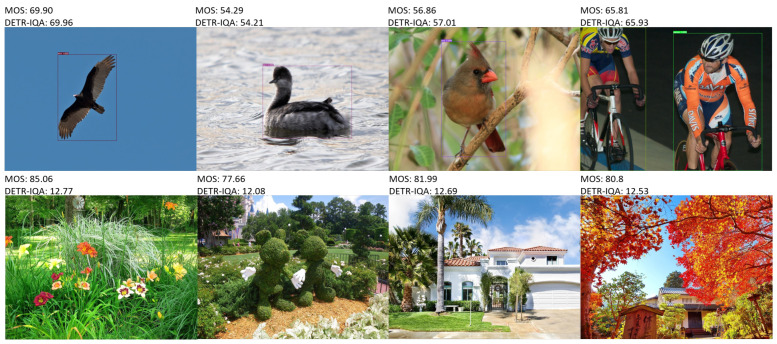
Some examples of images with MOS and a predicted score of DETR-IQA. Upper four images contain objects of interest and lower four images do not contain objects of interest.

We used $AP_{50}$, $AP_{75}$, $AP_{S}$, $AP_{M}$, and $AP_{L}$ to comprehensively measure object detection performance, and PLCC and SRCC were used to measure the image quality prediction accuracy of the model relative to the test dataset (represented by T) and images without objects of interest (represented by NO). Some examples of images with ground-truth MOS and the predicted score of DETR-IQA are illustrated in Figure 5, and it can be observed that DETR-IQA can carry out reasonable and accurate image quality assessment.

Finally, we calculated the FLOPs and FPS of DETR and DETR-IQA to compare their computation and complexity, and the results are shown in Table 5.

## 4. Discussion

In this study, we attempted to solve the practical problem of blindly assessing the quality of images containing objects of interest in some real-world applications. Thus, we selected the largest-scale authentic dataset KonIQ-10k [54] as the base dataset. Since the crowd workers were only instructed to consider the distortion of the entire image, the subjective quality score they provided was not closely related to the objects in the images. In order to solve this problem, we first predefined five classes—person, bird, dog, horse, and other animals (such as sheep and monkey)—and manually extracted the images containing objects belonging to these classes. To ensure that the subjective score is closely related to the objects of interest, we extracted images in which the objects of interest were the main content component in order to construct the experimental dataset. As shown in Table 1, the extracted images exhibited a long-tailed distribution, in which the number of images containing a person was close to 60% and the proportion of images containing other classes of objects comprised a smaller portion; real-world datasets typically exhibit this imbalanced distribution [57]. The consistency with the distribution of the real-world dataset indicated that the constructed dataset could be used to simulate the data generated by real-world scenarios, such as camera traps used to monitor wild animals. To test whether DETR-IQA has the ability to carry out reasonable and accurate quality assessments on images without objects of interest, we also manually extracted images without the above classes of objects from the KonIQ-10k dataset. We used 10 intervals as the MOS segment, and we counted the number of extracted images in each MOS segment. As shown in Figure 4, most images in the two extracted images datasets have an MOS between 50 and 80, and the number of images accounts for more than 70%. There are very few images in the two extracted image datasets with an MOS that is lower than 20, and the number of images does not exceed 2%. The MOS distribution of both datasets is consistent with the MOS distribution of the entire KonIQ-10k dataset.

In this study, we focus on a practical problem in the field of blind image quality assessment: In real-world applications, for the purpose of monitoring certain objects, the quality of images should be better than that of images without objects of interest even if the former images exhibit more severe distortion than the latter images because the images containing objects record important information—such as the activity and behavior of objects. However, according to the current methods, the quality assessment produced opposite results. Ignoring the distortion of the image region of objects of interest may lead to inconsistency between the predicted quality and human visual perception because the human visual system is sensitive to the visual quality of objects of interest in an image. Based on the assumption that people are more concerned about the visual quality of objects of interest, we added a simple multi-layer perceptron on the top of the transformer decoder of DETR to regress the feature of objects onto the quality score. Because of the surrounding auxiliary information relative to objects, we also simply took advantage of the feature of no-objects to assist in quality prediction. The proposed end-to-end model, DETR-IQA, possesses the capability to simultaneously carry out object detection and blind image quality assessment. Thus, the performance evaluation should comprehensively consider object detection performance and image quality assessment accuracy. In DETR-IQA, the image quality assessment and object detection shared the same original DETR architecture. The quality evaluation score not only did not contain any information, such as location, that could help improve the performance of object detection, but the quality evaluation head could also increase the loss of object detection and degrade the performance results. However, DETR-IQA carried out quality assessment based on the results of DETR. Therefore, the object detection performance was vital. We conducted baseline object detection experiments using DETR without IQA heads, and the baseline object detection results are shown in Table 3. We designed the loss of IQA heads with hyperparameter lambda to simply divide the MOS linearly in order to map the features of objects with the auxiliary features of noobjects to obtain the subjective quality score. We conducted several experiments using DETR-IQA with different λ—0.99, 0.9, 0.8, 0.7, and 0.6—and the results, including the object detection performance and image quality assessment accuracy, are shown Table 4. Comparing the five types of AP in Table 3 and Table 4, the various declining trends in object detection can be observed when hyperparameter lambda is set to different values. When λ was 0.8, the performance of object detection degraded the least. At the same time, the IQA accuracy was the best relative to the test set and image set without objects of interest. This result shows that, to some extent, the performance of object detection determines the accuracy of the image quality assessment. When λ was set to 0.99, 0.9, 0.7, and 0.6, the results indicated that the feature of no objects can provide auxiliary but limited information for image quality assessments. A λ value of 0.8 also indicated that the predicted quality score of an image without objects of interest would not exceed 20 due to the absence of objects of interest. As shown in Figure 4, the number of images with an MOS below 20 did not exceed 2% in the two extracted images datasets; this is, again, relative to the entire KonIQ-10k dataset. When λ was set to 0.8, DETR-IQA guaranteed that the predicted quality score of images containing objects of interest was generally greater than that of images without objects of interest unless the former had particularly severe distortion. Moreover, according to the results of PLCC and SRCC in the last two columns of Table 3, the quality assessment scores of DETR-IQA for images without objects of interest are highly correlated with subjective scores. This indicates that DETR-IQA can carry out reasonable and accurate image quality score predictions. Some examples of images with MOS and the predicted score of DETR-IQA in Figure 5 show that DETR-IQA can produce accurate quality assessments of images containing objects of interest and reasonable quality assessments of images without objects of interest even if the MOS of images containing objects of interest is lower than that of images without objects of interest; moreover, the predicted quality scores are highly related to the distortion of images.

We investigated the performance of methods proposed in the other blind IQA research literature on the entire KonIQ-10k dataset. The existing methods adopted many advanced architectures and tricks to fit the predicted score and the ground-truth subjective score, MOS, on the KonIQ-10k dataset. Moreover, the MOS only functions in accordance with the distortion of an entire image without considering the objects of interest. The dataset constructed in this study only ensures that MOS is mainly based on the objects in the image. Even so, the performance of our proposed DETR-IQA method with respect to predicting image quality exceeded that of some deep models that are specially designed for only performing image quality assessments, such as MEON [42] and CNN [23]. Moreover, our proposed DETR-IQA has the advantage of avoiding false quality assessments in which the predicted quality score of images containing objects of interest is lower than that of images without objects of interest. The results in Table 5 show that the proposed method negligibly increased the calculation and complexity of the DETR model and did not decrease inference speeds. If applied in practical applications, the proposed DETR-IQA method can simultaneously perform object detection and IQA via multi-tasking, which can simplify the conventional process—in which object detection is first carried out, followed by image quality assessment—and greatly reduce the workload.

Finally, in this study, the accuracy of DETR-IQA in image quality assessments was constrained by the performance of object detection. Since the original DETR has drawbacks such as slow convergence and instability, we leveraged SOTA adaptive activation functions [58] to accelerate the convergence and stabilize the performance of DETR-IQA. In addition, other models with better detection performances, such as Deformable-DETR [59], DINO [60], Co-DETR [61], etc., can be used to extract the features of objects more accurately, thus improving the accuracy of IQA. Moreover, the IQA heads in this study only used a very simple multi-layer perceptron to achieve better results than some deep learning-based models. Therefore, the design and use of more complex and advanced IQA heads, such as SwinTransformer [62], and the combination of CNN and transformer, could improve IQA accuracy.

## 5. Conclusions

In some practical image applications, images containing objects of interest are more valuable than images without objects of interest, even though the former exhibit greater distortion. Therefore, humans should be more concerned with the distortion of objects of interest. In this paper, the proposed model, DETR-IQA, extended DETR using IQA heads to simulate how humans perceive the quality of images containing objects of interest. The model can blindly predict the image quality score based on the features of objects detected using DETR, and the object detection performance only exhibits a slight decrease. The IQA heads consisting of very simple multi-layer perceptron architecture can not only accurately and blindly assess the quality of images containing objects of interest but also perform reasonable quality assessments on images without objects of interest. With the negligible increase in the model’s computation and the complexity of object detection, DETR-IQA can perform object detection and IQA simultaneously. DETR-IQA is a simple yet meaningful effort toward solving the practical IQA problem. In the future, we will use more advanced object detection networks and design more powerful IQA heads to improve their potential in practical image applications.

## Figures and Tables

**Table 1:** The details of images containing five classes of objects of interest.

Class	No. of Images
Person	2069
Bird	543
Dog	456
Horse	105
Other animal	541

**Table 2:** The extracted images’ training set and testing set assignments.

Training Set	Test Set
2866	716

**Table 3:** The results of DETR based on the constructed dataset.

Model Name	${\mathrm{AP}}_{50}$	${\mathrm{AP}}_{75}$	${\mathrm{AP}}_{S}$	${\mathrm{AP}}_{M}$	${\mathrm{AP}}_{L}$
DETR	0.74	0.663	0.112	0.269	0.678

Note: $AP_{50}$ is the average precision calculated when IoU is 0.5; $AP_{75}$ is the average precision calculated when Iou is 0.75; $AP_{S}$ is the average precision calculated when the object is small; $AP_{M}$ is the average precision calculated when the object is sized in the middle; $AP_{L}$ is the average precision calculated when object is large.

**Table 4:** The results of several DETR-IQA experiments with different hyperparameters, λ.

λ	${\mathrm{AP}}_{50}$	${\mathrm{AP}}_{75}$	${\mathrm{AP}}_{S}$	${\mathrm{AP}}_{M}$	${\mathrm{AP}}_{L}$	${\mathrm{PLCC}}_{T}$	${\mathrm{SRCC}}_{T}$	${\mathrm{PLCC}}_{\mathrm{NO}}$	${\mathrm{SRCC}}_{\mathrm{NO}}$
0.99	0.728	0.646	0.118	0.21	0.657	0.746	0.707	0.282	0.299
0.90	0.726	**0.647**	**0.224**	0.201	0.665	0.732	0.686	0.549	0.514
0.80	**0.741**	**0.647**	0.114	0.267	**0.668**	**0.785**	**0.727**	**0.659**	**0.609**
0.70	0.73	0.642	0.083	0.256	0.663	0.763	0.721	0.643	0.591
0.60	0.672	0.589	0.107	**0.299**	0.61	0.735	0.709	0.645	0.565

Note: $PLCC_{T}$ and $SRCC_{T}$ are, respectively, the Pearson and Spearman correlation coefficients relative to the test set; $PLCC_{NO}$ and $SRCC_{NO}$ are, respectively, the Pearson and Spearman correlation coefficients of the images without objects of interest.

**Table 5:** The results of FLOPs and the FPS of DETR and DETR-IQA.

Model Name	FLOPs	Inference FPS	Params
DETR-IQA	70.09 G	41.41 M	20
DETR	70.01 G	41.27 M	20

## Data Availability

The public KonIQ-10k dataset link: https://osf.io/hcsdy/.
